# Supplementary material for: Deubiquitinating Enzymes Ubiquitin-Specific Proteases 7 and 10 Regulate TAU Aggregation
Source: Int J Mol Sci. 2025 Nov 15;26(22):11062. doi: 10.3390/ijms262211062 (PMC12652193; doi:10.3390/ijms262211062)
Supplement: Supplementary file 1 [file ijms-26-11062-s001.zip › Volbracht_ suppl. figure legends_IJMS revision.pdf]

Supplementary Table 1. **List of antibodies used for immunocytochemistry (ICC) and Western blot analysis (WB)**

**Type**	**Antibody, catalogue number**	**Application, working dilution**	**Source**
Primary antibody	Mouse anti-GFAP, 53809	ICC, 1:1000	Novus Biologicals, Centennial, CO, USA
	Mouse anti-pS202/pT205 tau, clone AT8, MN1020	ICC, 1:500	Invitrogen, ThermoFisher Scientific, Waltham, MA, USA
	Rabbit anti-pS422 tau, 44764G	ICC, 1:500	Invitrogen, ThermoFisher Scientific, Waltham, MA, USA
	Rabbit anti-tau (Dako), A0024	ICC, 1:5000	Agilent, Santa Clara, CA, USA
	Mouse anti-NEFL, 13-0400	ICC, 1:200	Invitrogen, ThermoFisher Scientific, Waltham, MA, USA
	Mouse anti-ubiquitin, clone FK2, 04-263	ICC, 1:500	Merck Millipore, Darmstadt, Germany
	Mouse anti-ubiquitin, clone P4D1, sc8017	ICC, 1:500	Santa Cruz Biotechnology, Dallas, TX, USA
	Rabbit anti-human tau, E1	WB, 1:1000	[51]
	Mouse anti-pS396 tau, clone D1.2	WB, 1:1000	[41]
Secondary antibody	Alexa488 donkey anti-mouse, A21202	ICC, 1:1000	Invitrogen, ThermoFisher Scientific, Waltham, MA, USA
	Cy3 donkey anti-rabbit, 715-545-150	ICC, 1:1000	Jackson ImmunoResearch Europe Ltd, Ely, UK
	IRDye 680RD goat anti-mouse, 926-68070	WB, 1:5000	LICORbio, Lincoln, NE, USA
	IRDye 800RD goat anti-rabbit, 926-32211	WB, 1:5000	LICORbio, Lincoln, NE, USA

Supplementary figure legends

Supplementary Figure 1. **Seeded TAU aggregation displayed as AT8-positive TAU inclusions and as aggregated and hyperphosphorylated TAU species in CTX from rTg4510 mice.** A) Cortical neurons isolated from rTg4510 mouse embryos were incubated at days in vitro (DIV) 7 with 2 ng/µL S1p or P3 TAU seeds isolated from brains of 32-40 weeks old rTg4510 mice or untreated (naive), methanol fixed at DIV 15 and processed for immunocytochemistry. Hyperphosphorylated TAU was detected by AT8 antibody. Representative confocal images (20x objective) of nuclei stained with Hoechst (in gray) and of seeded TAU aggregation detected by AT8 immunoreactivity (in green). The scale bar corresponds to 50 µm. B) Cortical neurons isolated from non-

transgenic (non-tg) or rTg4510 mouse embryos were incubated at DIV 7 with 2 ng/µL S1p or P3 isolated from brains of 32-40 weeks old rTg4510 mice (S1p rTg4510 and P3 rTg4510) or untreated (naive), extracted into soluble Triton and insoluble SDS fractions at DIV 15 and analysed by western blot for total human TAU (E1) and phosphorylated tau at the S396 epitope (pS396-tau). The E1 antibody recognizes exclusively human TAU at non-phosphorylated and phosphorylated epitopes. Phosphorylated and non-phosphorylated human 0N4R transgene TAU was displayed at 55 kDa with the E1 antibody in the soluble Triton fraction. Phosphorylated human 0N4R transgene TAU was displayed at 55 kDa and phosphorylated murine Tau isoforms were displayed and 45 and 55 kDa with the pS396-tau antibody in the soluble Triton fraction. Hyperphosphorylated human 0N4R transgene TAU is mobility-shifted and was displayed at 64 kDa with the E1 and pS396-tau antibodies exclusively in insoluble SDS fractions of CTX from rTg4510 seeded with S1p or P3 rTg4510. Representative western blots from 3 independent experiments.

Supplementary Figure 2. **Inducers of autophagy reduce seeded TAU aggregation in CTX from rTg4510 mice.** Cortical neurons isolated from rTg4510 mouse embryos were incubated at DIV 7 with 2 ng/µL S1p TAU seeds isolated from brains of 32-40 weeks old rTg4510 mice, treated with trehalose or rapamycin at the indicated concentrations at DIV 8 and harvested at DIV 15 to measure seeded TAU aggregation by the TAU aggregation assay. Data are presented as percentage seeded TAU aggregation normalized to total protein, mean ± SD from 5 wells representative from 3 independent experiments. Dotted black lines indicate the negative control values for PBS and DMSO. Brown-Forsythe and Welch ANOVA with Dunnett's T3 post hoc test, asterisks indicate significance (** $p < 0.01$, *** $p < 0.001$, ****$p < 0.0001$).

Supplementary Figure 3. **Inter and intra plate variations and Z' values of the seeded TAU aggregation assay in CTX from rTg4510 mice.** A) Cortical neurons isolated from rTg4510 mouse embryos were incubated at DIV 7 with 2 ng/µL S1p TAU seeds isolated from brains of 32-40 weeks old rTg4510 mice and harvested at DIV 15 to measure seeded TAU aggregation by the TAU aggregation assay. Data are presented as percentage seeded TAU aggregation normalized to total protein, mean ± SD from 5 wells from the 3 independent plates and from each individual plate. B) Cortical neurons isolated from rTg4510 mouse embryos were incubated at the indicated time points (DIV 1, DIV 4, DIV 6) with 1 µM siMAPT or non-targeting control siRNA (neg. con), incubated at DIV 7 with 2 ng/µL S1p TAU seeds isolated from brains of 32-40 weeks old rTg4510 mice and harvested at DIV 15 to measure seeded TAU aggregation by the TAU aggregation assay. Data are presented as percentage seeded TAU aggregation normalized to total protein, mean ± SD from 5 wells for siMAPT and from 10 wells for non-targeting control siRNA (neg. con). Z' values of 0.66, 0.67, and 0.56 with siRNA incubation at DIV 1, DIV 4, and DIV 6, respectively, were calculated.

Supplementary Figure 4. **Accell smartpool siRNA targeting 93 mouse DUBs and plate layout of the DUB siRNA screening plates.** A) Cortical neurons isolated from rTg4510 mouse embryos were incubated with 1 µM of the listed smartpool siRNA targeting 93 mouse DUBs, non-targeting control siRNA or siMAPT in the primary screens. B) Illustration of the plate layout for the DUB siRNA screens.

Supplementary Figure 5. **Accell smartpool siRNA Usp7 and Usp10 reduces seeded TAU aggregation in CTX from rTg4510 mice.** Cortical neurons isolated from rTg4510

mouse embryos were incubated at DIV 1 (A) or DIV 6 (B) with 1 μM smartpool siRNA targeting mouse DUBs, MAPT, or non-targeting control (neg. con). At DIV 7, cultures were treated with 2 ng/μL S1p TAU seeds isolated from brains of 32-40 weeks old rTg4510 mice and harvested at DIV 15 to measure seeded TAU aggregation by the TAU aggregation assay. Data are presented as percentage seeded TAU aggregation normalized to total protein, mean ± SD from 5 wells and mean ± SD from 10 wells for neg. con. The dotted black line indicates the negative control value, and the dotted red lines indicate the ± 20% margins. A and B) Primary screen with the 93 DUB siRNAs. Brown-Forsythe and Welch ANOVA with Dunnett's T3 post hoc test, asterisks indicate significance (*$p < 0.05$, ** $p < 0.01$, *** $p < 0.001$, ****$p < 0.0001$).

Supplementary Figure 6. **Confirmation and validation of siRNA Usp7 and Usp10 reducing seeded TAU aggregation in CTX from rTg4510 mice.** Cortical neurons isolated from rTg4510 mouse embryos were incubated at DIV 1 (A) or DIV 6 (B) with 1 μM smartpool siRNA targeting mouse DUBs, MAPT, or non-targeting control (neg. con). At DIV 7, cultures were treated with 2 ng/μL S1p TAU seeds isolated from brains of 32-40 weeks old rTg4510 mice and harvested at DIV 15 to measure seeded TAU aggregation by the TAU aggregation assay. Data are presented as percentage seeded TAU aggregation normalized to total protein, mean ± SD from 5 wells and mean ± SD from 10 wells for neg. con. The dotted black line indicates the negative control value, and the dotted red lines indicate the ± 20% margins. A and B) Confirmation screens with the 32 DUB siRNA hits. C) Validation screen with 11 confirmed DUB siRNA hits incubated at DIV 1 using 2 ng/μL S1p or P3 TAU seeds at DIV 7. Brown-Forsythe and Welch ANOVA with Dunnett's T3 post hoc test, asterisks indicate significance (*$p < 0.05$, ** $p < 0.01$, *** $p < 0.001$, ****$p < 0.0001$).

Supplementary Figure 7. **Usp7 and Usp10 knockdown does not affect human TAU levels in CTX from rTg4510 mice.** Cortical neurons isolated from rTg4510 mouse embryos were incubated at DIV 1 with 1 μM smartpool (sp) or individual siRNAs (#13, #14, #15, and #16) targeting Usp7 (A) and Usp10 (B), MAPT, or non-targeting control (neg. con) and harvested to measure human TAU protein levels by ELISA at DIV 15 (A and B). Data are presented as bar graphs as means ± SD from 3 wells representative from 3 independent experiments and human TAU expression normalized to total protein. Brown-Forsythe and Welch ANOVA with Dunnett's T3 post hoc test, asterisks indicate significance (****$p < 0.0001$).

Supplementary Figure 8. **Silencing of Usp7 and Usp10 in OHSCs from rTg4510 mice.** Organotypic hippocampal slice cultures isolated from rTg4510 mouse pups were treated at DIV 0 with 1 μM siRNA Usp7 #13 or Usp10 #14 or non-targeting control and harvested at DIV 8 to measure messenger RNA levels by qPCR. Data are presented as bar graphs as means ± SD from 3 slices representative from 2 independent experiments with non-targeting control siRNA set to 1.
