# Supplementary material for: Deubiquitinating Enzymes Ubiquitin-Specific Proteases 7 and 10 Regulate TAU Aggregation
Source: Int J Mol Sci. 2025 Nov 15;26(22):11062. doi: 10.3390/ijms262211062 (PMC12652193; doi:10.3390/ijms262211062)
Supplement: Supplementary file 1 [file ijms-26-11062-s001.zip › Volbracht_suppl. figures_IJMS revision.pdf]

Fig S1

A

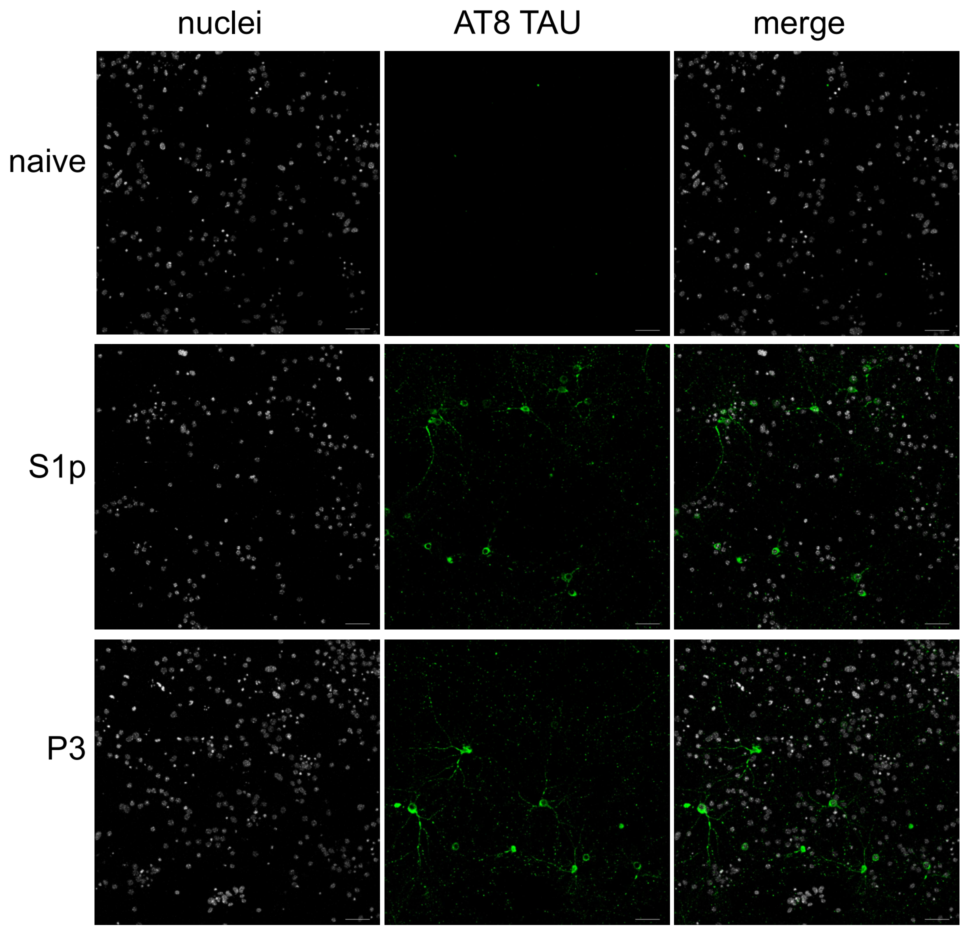
nuclei
AT8 TAU
merge
naive
S1p
P3

B

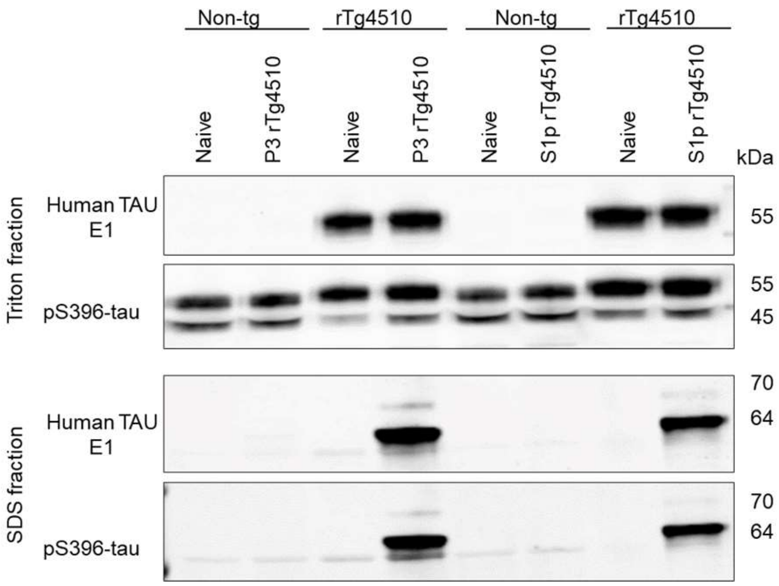
Non-tg
rTg4510
Non-tg
rTg4510
Naive
P3 rTg4510
Naive
P3 rTg4510
Naive
S1p rTg4510
Naive
S1p rTg4510
kDa
Triton fraction
Human TAU E1
55
pS396-tau
55
45
SDS fraction
Human TAU E1
70
64
pS396-tau
70
64

Fig S2

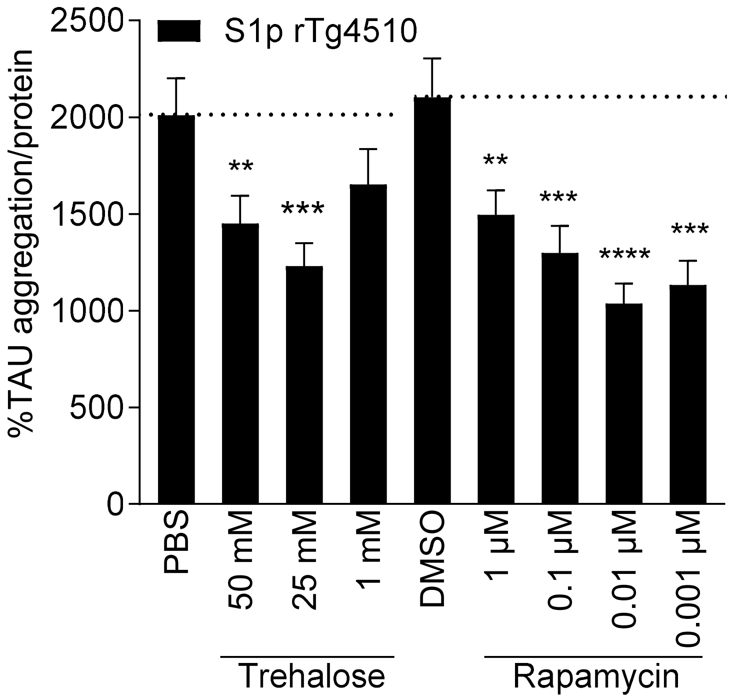

S1p rTg4510
%TAU aggregation/protein
2500
2000
1500
1000
500
0
**
***
**
***
****
***
PBS
50 mM
25 mM
1 mM
DMSO
1 µM
0.1 µM
0.01 µM
0.001 µM
Trehalose
Rapamycin

Fig S3

A

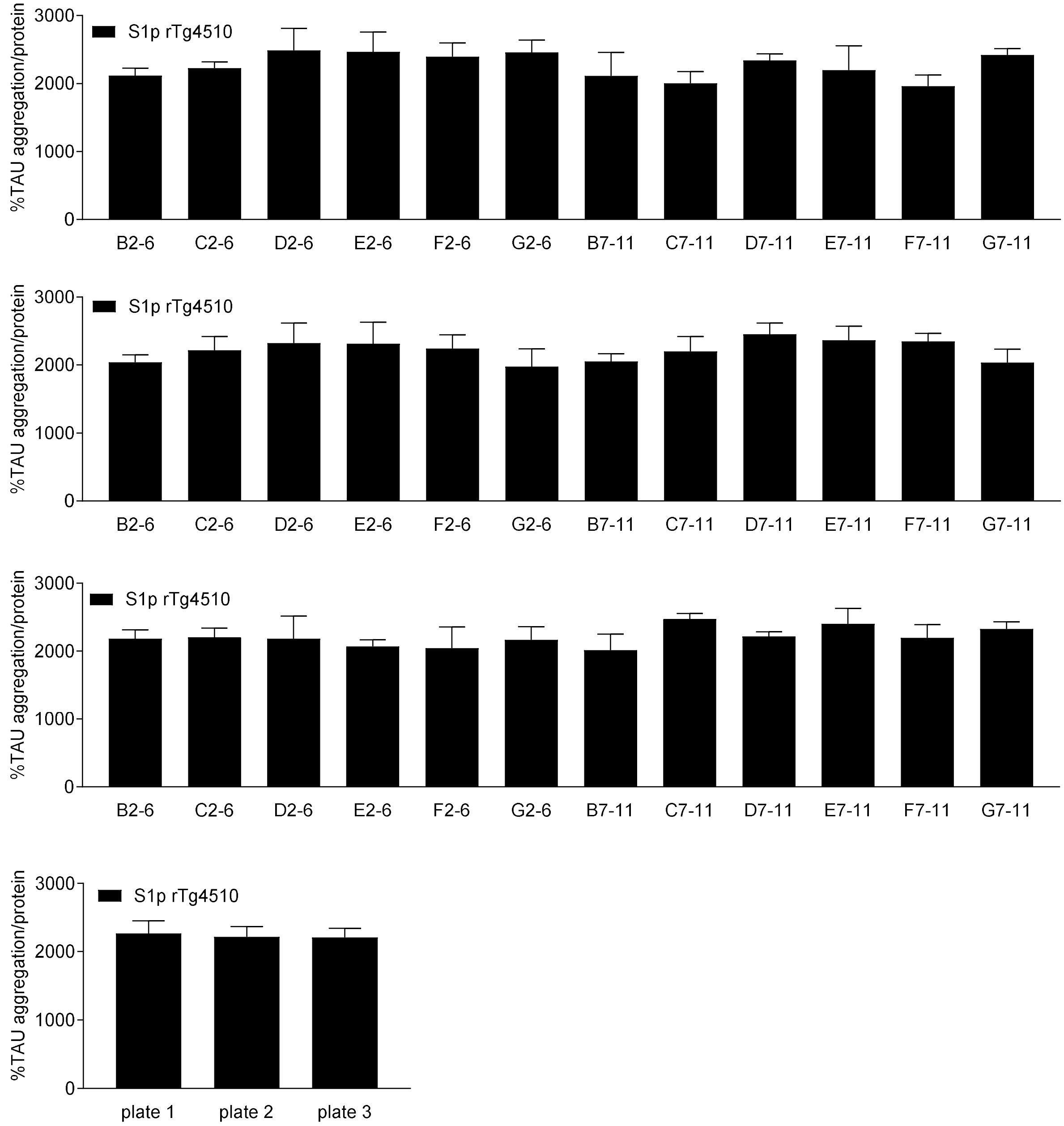

%TAU aggregation/protein
S1p rTg4510
3000
2000
1000
0
B2-6
C2-6
D2-6
E2-6
F2-6
G2-6
B7-11
C7-11
D7-11
E7-11
F7-11
G7-11
plate 1
plate 2
plate 3

B

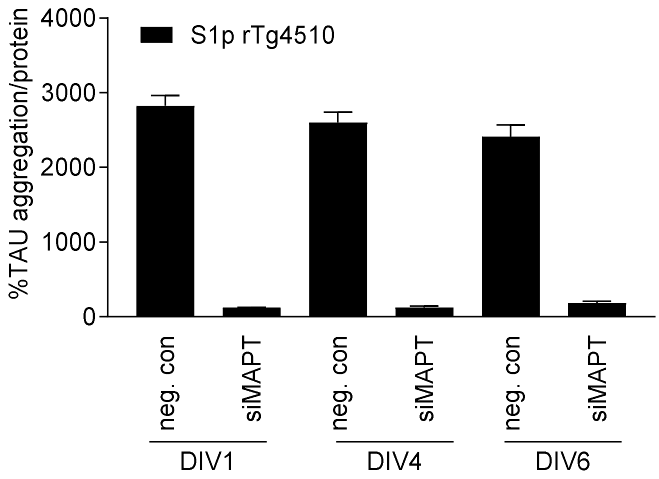

%TAU aggregation/protein
S1p rTg4510
4000
3000
2000
1000
0
neg. con
siMAPT
DIV1
DIV4
DIV6

Fig S4

A

Usp1	Usp10	Usp20	Usp30	Usp39	Usp49	Stambp	Eif3h	Otud6a	Uchl5	Senp5
Usp2	Usp11	Usp21	Usp31	Usp40	Usp50	Stambpl1	Prpf8	Otud6b	Bap1	Senp6
Usp3	Usp12	Usp22	Usp32	Usp42	Usp51	Cops5	Otub1	Otud7a	Atxn3	Senp7
Usp4	Usp13	Usp24	Usp33	Usp43	Pan2	Cops6	Otub2	Otud7b	Josd1	
Usp5	Usp14	Usp25	Usp34	Usp44	Usp53	Brcc3	Otud1	Tnfaip3	Josd2	
Usp7	Usp15	Usp26	Usp35	Usp45	Usp54	Mpnd	Yod1	Zranb1	Taf1d	
Usp8	Usp16	Usp27x	Usp36	Usp46	Cyld	Mysm1	Otud3	Vcpip1	Senp1	
Usp9x	Usp18	Usp28	Usp37	Usp47	Uspl1	Psmd7	Otud4	Uchl1	Senp2	
Usp9y	Usp19	Usp29	Usp38	Usp48	Psmd14	Eif3f	Otud5	Uchl3	Senp3	

B

	1	2	3	4	5	6	7	8	9	10	11	12
A												
B		siRNA buffer					siRNA Dub5					
C		siRNA Dub1					siRNA non-targeting control					
D		siRNA Dub2					siRNA Dub6					
E		siRNA non-targeting control					siRNA Dub7					
F		siRNA Dub3					siRNA Dub8					
G		siRNA Dub4					siMAPT					
H												

Fig S5

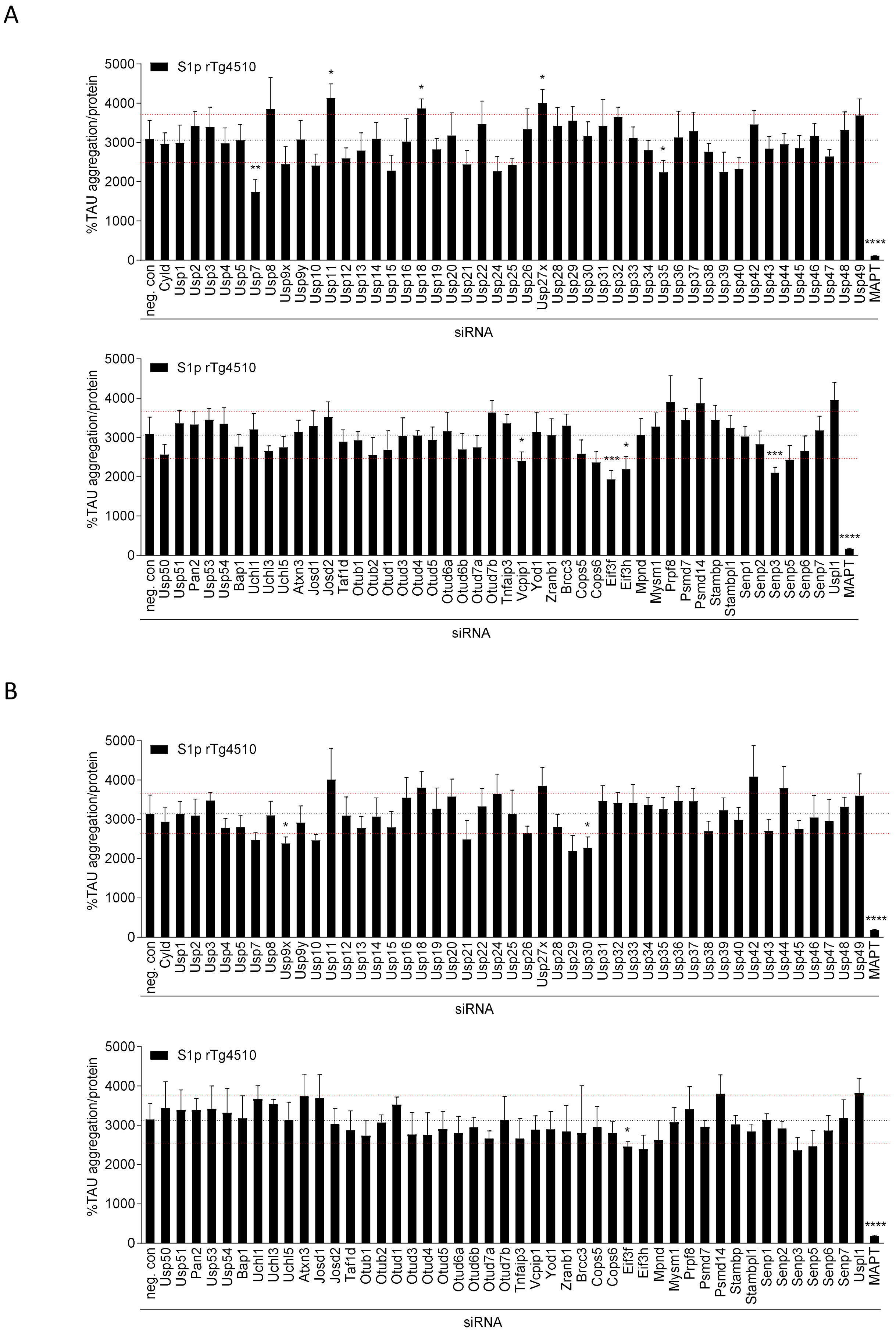
A
S1p rTg4510
%TAU aggregation/protein
0
1000
2000
3000
4000
5000
neg. con
Cyld
Usp1
Usp2
Usp3
Usp4
Usp5
Usp7
Usp8
Usp9x
Usp9y
Usp10
Usp11
Usp12
Usp13
Usp14
Usp15
Usp16
Usp18
Usp19
Usp20
Usp21
Usp22
Usp24
Usp25
Usp26
Usp27x
Usp28
Usp29
Usp30
Usp31
Usp32
Usp33
Usp34
Usp35
Usp36
Usp37
Usp38
Usp39
Usp40
Usp42
Usp43
Usp44
Usp45
Usp46
Usp47
Usp48
Usp49
MAPT
siRNA
Usp50
Usp51
Pan2
Usp53
Usp54
Bap1
Uchl1
Uchl3
Uchl5
Atxn3
Josd1
Josd2
Taf1d
Otub1
Otub2
Otud1
Otud3
Otud4
Otud5
Otud6a
Otud6b
Otud7a
Otud7b
Tnfaip3
Vcpip1
Yod1
Zranb1
Brcc3
Cops5
Cops6
Eif3f
Eif3h
Mpnd
Mysm1
Prpf8
Psmd7
Psmd14
Stambp
Stambpl1
Senp1
Senp2
Senp3
Senp5
Senp6
Senp7
Uspl1
B

Fig S6

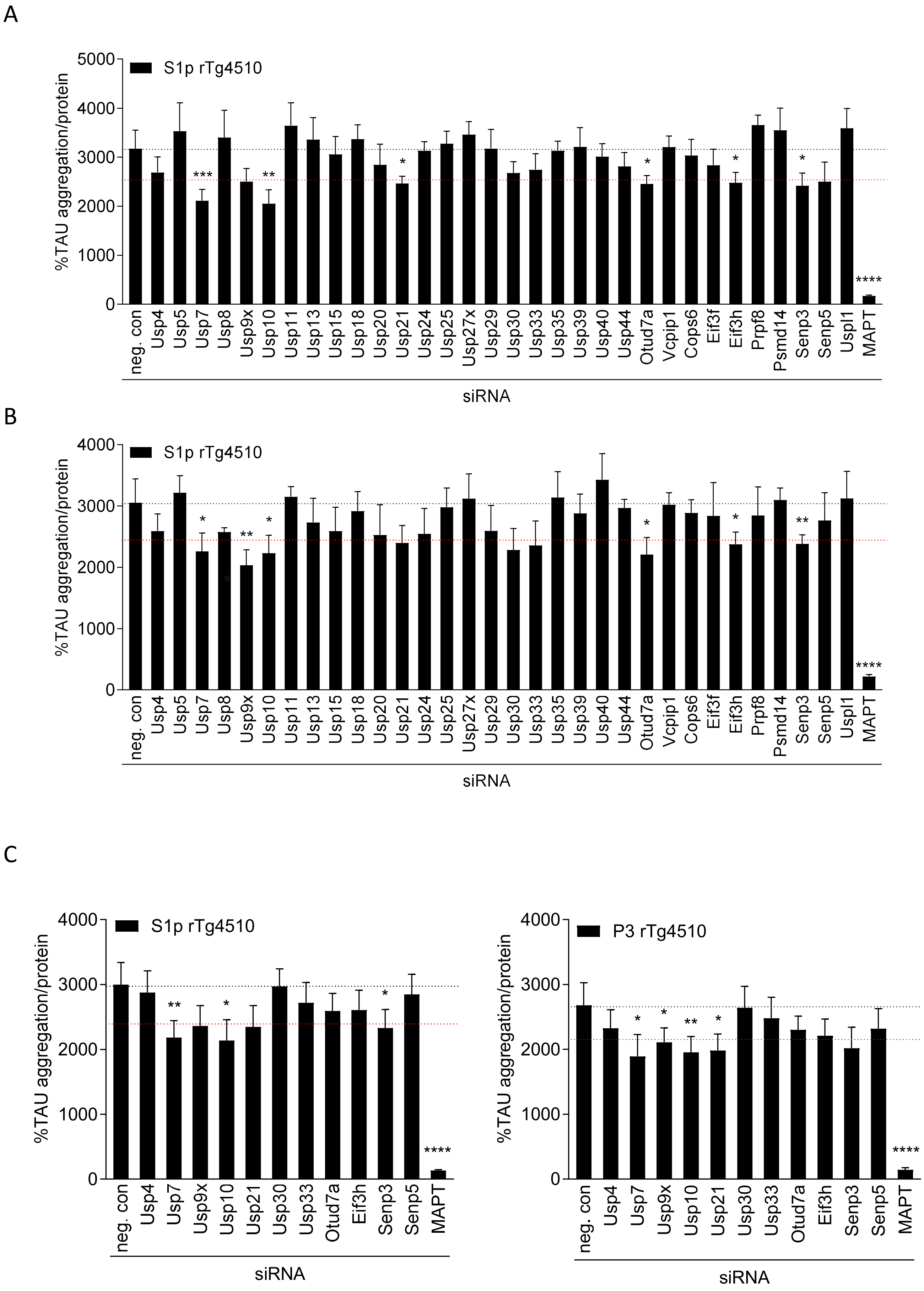
A
B
C
S1p rTg4510
P3 rTg4510
%TAU aggregation/protein
siRNA
neg. con
Usp4
Usp5
Usp7
Usp8
Usp9x
Usp10
Usp11
Usp13
Usp15
Usp18
Usp20
Usp21
Usp24
Usp25
Usp27x
Usp29
Usp30
Usp33
Usp35
Usp39
Usp40
Usp44
Otud7a
Vcpip1
Cops6
Eif3f
Eif3h
Prpf8
Psmd14
Senp3
Senp5
Uspl1
MAPT
0
1000
2000
3000
4000
5000
*
**
***
****

Fig S7

A

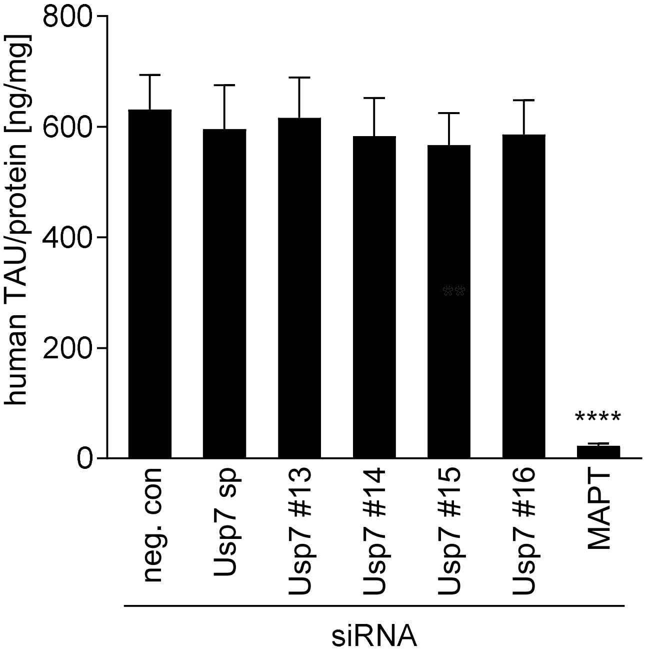

800
600
400
200
0
human TAU/protein [ng/mg]
****
neg. con
Usp7 sp
Usp7 #13
Usp7 #14
Usp7 #15
Usp7 #16
MAPT
siRNA

B

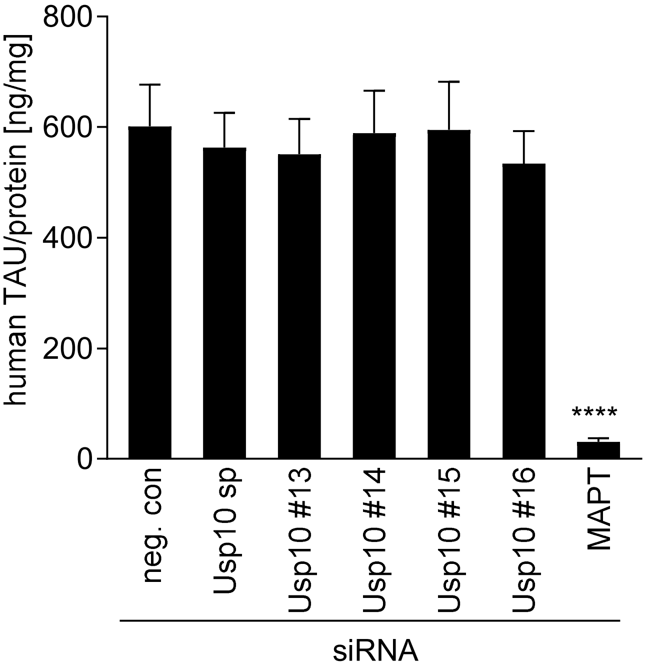

800
600
400
200
0
human TAU/protein [ng/mg]
****
neg. con
Usp10 sp
Usp10 #13
Usp10 #14
Usp10 #15
Usp10 #16
MAPT
siRNA

Fig. S8

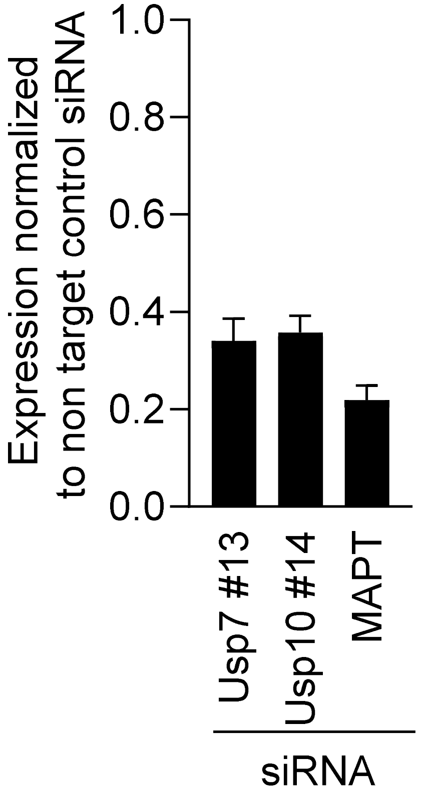

Expression normalized
to non target control siRNA
1.0
0.8
0.6
0.4
0.2
0.0
Usp7 #13
Usp10 #14
MAPT
siRNA
